# Efficacy and safety of small-incision corneal intrastromal lenticule implantation for hyperopia correction: a systematic review and meta-analysis

**DOI:** 10.3389/fmed.2024.1320235

**Published:** 2024-02-16

**Authors:** Yue Wang, Jingjing Zheng, Zuofeng Guo, Xuejun Fang

**Affiliations:** ^1^Ophthalmology, Liaoning Aier Eye Hospital, Shenyang, China; ^2^AIER School of Ophthalmology, Central South University, Changsha, China

**Keywords:** lenticule implantation, hyperopia, small incision lenticule extraction, refractive, meta-analysis

## Abstract

**Purpose:**

To assess the efficacy and safety of intrastromal lenticule implantation for the treatment of hyperopia.

**Methods:**

A systematic search of PubMed, Web of Science, Embase, Cochrane Library, China National Knowledge Internet, and Wan Fang Database identified studies on small-incision intrastromal lenticule implantation for hyperopia correction until January 2023. The Joanna Briggs Institute (JBI) critical appraisal tool was used to assess the quality of the retrospective research, and the Methodological Index for Non-randomized Studies (MINORS) was used to assess the quality of the prospective research. This study included postoperative visual outcomes, corneal morphology, and biomechanical outcomes.

**Results:**

A total of 456 articles were identified, of which 10 were included in the meta-analysis. Ten single-arm studies involving 190 eyes were included. A meta-analysis demonstrated that corneal intrastromal lenticule implantation treatment significantly improved hyperopia. Uncorrected distance visual acuity (UDVA) significantly improved compared to the preoperative value (*p* = 0.027), corrected distance visual acuity showed no difference compared to the preoperative value (*p* = 0.27), and 87% eyes have no loss of one or more lines in the Snellen lines of CDVA (*p* < 0.00001). There was a significant difference between the spherical equivalent refractive (SE) and preoperative examination (*p* < 0.00001), 52% of eyes had ±0.5 diopters (D) postoperative SE (*p* < 0.00001), and 74% eyes had ±1.0 D postoperative SE (*p* < 0.00001). The central corneal thickness (CCT) increased by 72.68 μm compared to that preoperatively (*p* < 0.00001), and corneal curvature increased by 4.18D (*p* < 0.00001). The *Q*-value decreased by 0.82 (*p* < 0.00001), and higher-order aberration (HOA) decreased by 0.66 (*p* < 0.00001).

**Conclusion:**

Small-incision intrastromal lenticule implantation may be an effective solution for correcting hyperopia. The effect of improved vision is significant, but further exploration is needed for changes in corneal biomechanics and long-term safety.

**Systematic review registration**: https://www.crd.york.ac.uk/PROSPERO/, identifier: CRD42023432343.

## 1 Introduction

Hyperopia not only leads to blurred vision, but also poses a risk factor for diseases such as strabismus and bilateral amblyopia (1). For patients who want to correct hyperopia through refractive surgery, transepithelial photorefractive keratectomy (T-PRK), femtosecond laser-assisted *in-situ* keratomileusis (FS-LASIK) and small incision lenticule extraction (SMILE) can be used for the correction of hyperopia. These surgical methods can effectively correct hyperopia and ametropia; however, they all increase the risk of corneal swelling, refractive regression, and corneal epithelial implantation after surgery (2–4).

SMILE produces a corneal intrastromal lenticule with a diameter of 6–7 mm and a thickness of typically 30–130 μm while correcting myopia. Currently, there are reports of the application of corneal intrastromal lenticule in the treatment of diseases such as keratoconus (KC), corneal ulcers, corneal dilation caused by corneal refractive surgery, and corneal infection perforation (5–8). In addition, corneal intrastromal lenticule implantation has also been applied to the surgical treatment of hyperopia, and the postoperative effects are significant (9, 10). The long-term safety of corneal intrastromal lenticule implantation for hyperopia has not been confirmed, and owing to difficulties in preserving corneal intrastromal lenticule, it has not yet been widely approved for clinical use.

More recently, the number of SMILE surgeries has rapidly increased worldwide, and research on the preservation and reuse of corneal intrastromal lenticules is also becoming more mature. Corneal intrastromal lenticules can be preserved using various methods, including ultra-low temperature freezing, glycerol preservation, and commercial cryopreservation solutions (11, 12). This study conducted a systematic review of clinical studies on the treatment of hyperopia with corneal intrastromal lenticule implantation, and a meta-analysis of postoperative indicators such as UDVA, CDVA, CCT, corneal curvature, HOAs, and changes in corneal biomechanics. The aim was to explore the effectiveness and safety of corneal intrastromal lenticule implantation in the treatment of hyperopia, and to provide new treatment ideas for corneal refractive surgery for clinical hyperopia refractive errors.

## 2 Methods

We performed a systematic review and meta-analysis in accordance with the Reporting Items for Systematic Reviews and Meta-Analyses Statement (13). This study was registered in the International Prospective Register of Systematic Reviews (PROSPERO; ID: CRD42023398935). The study adhered to the tenets of the Declaration of Helsinki.

### 2.1 Search strategy

Two researchers (YW and JZ) independently performed database searches of PubMed, EMBASE, Web of Science, the Cochrane Library, China National Knowledge Internet, and the Wanfang Database to identify relevant studies on small-incision corneal intrastromal lenticule implantation for hyperopia correction up to 2023.01.01. The search terms included “small-incision intrastromal lenticule,” “lenticule implantation,” and “hyperopia.” The search strategy was determined after multiple pre-searches, combined with subject headings and free words.

### 2.2 Inclusion and exclusion criteria

The inclusion criteria were as follows: (1) the research subjects are hyperopia patients who have undergone small-incision corneal intrastromal lenticule implantation surgery. (2) For single-arm studies, the treatment modality in the included studies was corneal intrastromal lenticule implantation. For case-control studies, the experimental group comprised eyes treated with corneal intrastromal lenticule implantation, and there were no limitations on the control group intervention measures. (3) Studies that reported at least one major outcome (UDVA, CDVA, SE, and CCT) or secondary outcome (corneal biomechanical indicators and HOAs). (4) The patients included in the study had no other eye diseases. (5) Complete at least three months of follow-up.

The exclusion criteria were as follows: (1) the included patients are not those who have undergone small incision matrix lens transplantation surgery. (2) Lack of standard deviation in the research results. (3) No outcome was related to the purpose of the study. (4) Data with significant errors in research results. (5) Studies with fewer than five eyes were included. (6)Research involving duplicate patients.

The study was independently reviewed by two researchers (YW, JZ) to determine whether the included studies met the inclusion or exclusion criteria. When there was any disagreement, a third researcher (ZG) participated.

### 2.3 Data extraction

For all included studies, the basic characteristics of the article and the main clinical data were extracted. The basic characteristics of the article included: the lead author, year of publication, language of publication, sample size of patients and eyes, age, surgical method, and postoperative outcomes.

The primary outcome measures were visual outcome, refractive outcome, and corneal morphology change. The visual outcome included the mean logMAR value of uncorrected distance visual acuity (UDVA), mean logMAR value of corrected distance visual acuity (CDVA), and the eyes changes in the Snellen chart of CDVA. The refractive outcome included the mean postoperative spherical equivalent (SE), the percentage of eyes within ±0.5 diopters (D) of the target refraction and the percentage of eyes within ±1.0 D. The corneal morphology change outcome included the mean increment of corneal curvature and changes in central corneal thickness (CCT).

Secondary outcomes included postoperative high-order aberrations (HOAs), changes in *Q* values, and corneal biomechanical indicators. Among them, corneal biomechanical indicators include Corneal compensated intraocular pressure (IOPcc), Goldmann correlated intraocular pressure (IOPg), corneal hysteresis (CH), and corneal resistance factor (CRF).

Data were extracted independently by two authors (YW and JZ), and any differences were resolved through discussion until consensus was reached or a third author (ZG) is consulted.

### 2.4 Assessment of risk of bias

The Joanna Briggs Institute (JBI) critical appraisal tool was used to evaluate the methodological quality of retrospective studies (14, 15), and the Methodological Index for Non-randomized Studies (MINORS) was used to evaluate the methodological quality of prospective studies (16, 17). Studies were not included in our analysis if they scored lower than 6 out of 8 (75%) in retrospective studies, the scores of prospective studies must be greater than 10, and two reviewers (YW and JZ) independently evaluated the quality of the include studies. If there was a disagreement, another reviewer (ZG) participated in the discussion to obtain the results. Funnel plot and Egger’s test were used to evaluate the risk of publication bias, with *p* < 0.05 indicating a statistically significant bias.

### 2.5 Statistical analysis

Statistical analysis was conducted using Stata V.16.0 software (StataCorp, College Station), and forest maps were created using RevMan software (version 5.4.1; Cochrane Collaboration). In this meta-analysis, continuous variables were extracted as mean and standard deviation (mean ± SD), and estimated using the weighted mean difference (WMD), and 95% confidential intervals (CI). Random-effects models were used when study heterogeneity was high (*I*^2^ > 50%), and fixed-effects models were used when heterogeneity was low (*I*^2^ ≤ 50%) (18, 19).

## 3 Results

### 3.1 Study selection

**Figure 1:**
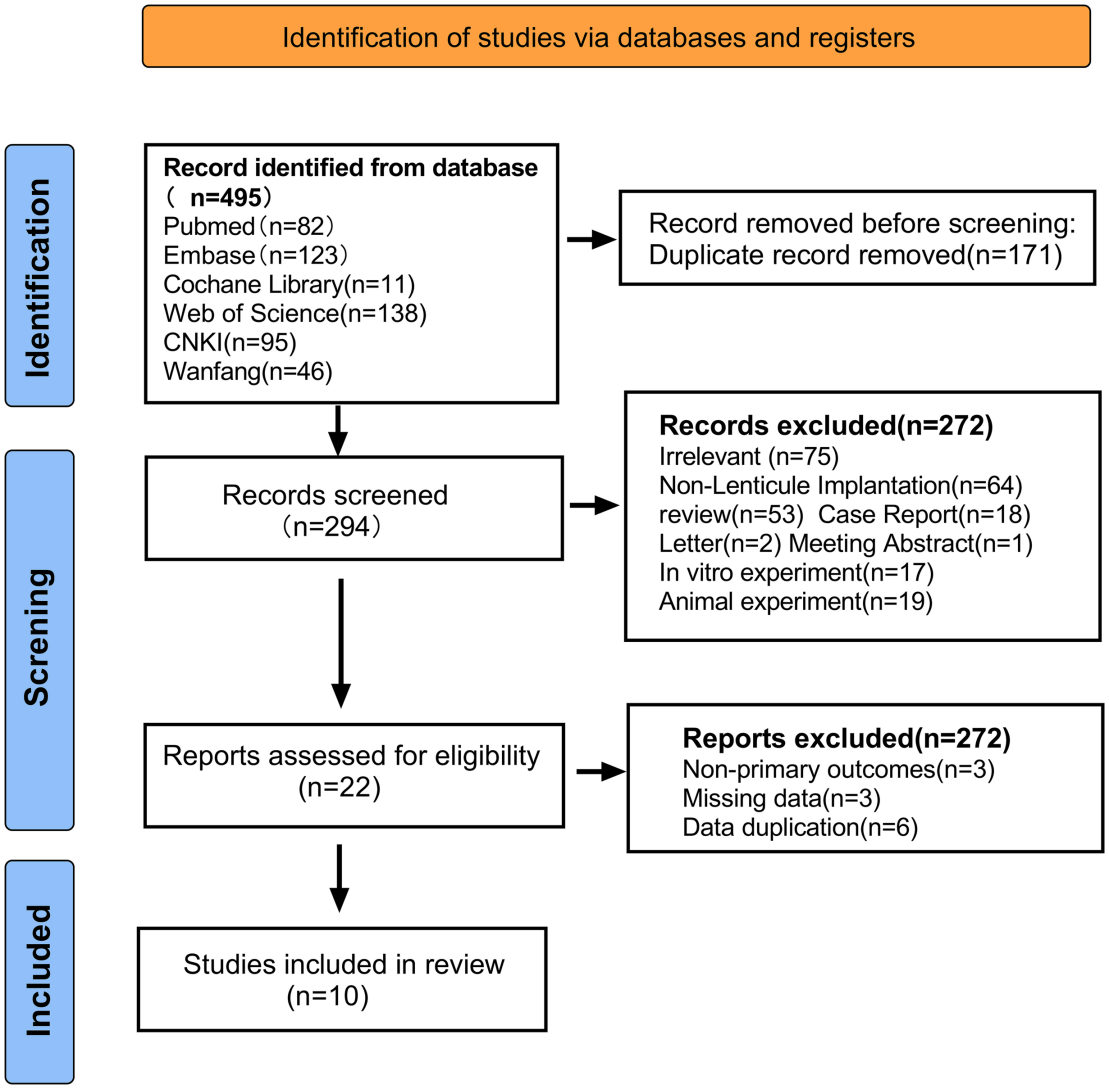
Preferred reporting items for systematic reviews and meta-analyses flow diagram of study selection.

We identified 465 related articles through a preliminary search, of which 171 duplicate articles were excluded using EndNote X9 software (Clarivate Analytics, US), and 294 articles underwent title and abstract reviews. After selecting titles and abstracts, 272 studies were excluded for the following reasons: irrelevant topics (*n* = 75), inconsistent research contents (*n* = 64), reviews (*n* = 53), case reports (*n* = 18), *in vitro* experimental studies (*n* = 17), animal studies (*n* = 39), literature corrections (*n* = 2), letters (*n* = 2), and conference literature (*n* = 1). A total of 22 articles were reviewed, of which 12 were excluded for the following reasons: no major outcome (*n* = 3), missing data (*n* = 3), and duplicate data (*n* = 6). The remaining 10 reports met the qualification criteria and were included in the meta-analysis (9, 10, 20–27). The literature screening process is illustrated in Figure 1.

### 3.2 Study characteristics

A total of 130 (190 eyes) cases were included in the study, with an average age of 24.98 (95% CI: [22.51, 27.44]). The characteristics of the included studies are summarized in Table 1.

**Table 1:** The basic clinical characteristics of included studies.

Study	Language	Design	Eyes	Surgical	Lenticule Implantation	Age	Outcomes
		single-arm study			Autologous/Allogeneic	Mean ± SD	
Jing Zhang (10)	English	Retrospective	24	SMILE	Allogeneic	26.40 ± 5.82	UDVA, CDVA, SE, HOAs, CCT, IOPg, IOPcc, CH, CRF, Km, Q value
Jie Hou (20)	English	Retrospective	31	SMILE	Allogeneic	20.00 ± 1.48	UDVA, SE, CD, CCT, Km
Meng Li (23)	English	Prospective	10	LASIK	Autologous	24.70 ± 5.82	CDVA, SE
Jiawei Wu (21)	English	Prospective	10	LASIK/PTK	Allogeneic	22.80 ± 3.29	CDVA, SE, Q value, HOAs
Ling Sun (24)	English	Prospective	5	LASIK	Autologous	24.60 ± 5.30	UDVA, CDVA, SE, Km, CCT
Shengtao Liu (26)	English	Prospective	5	SMILE	Allogeneic	21.00 ± 2.60	UDVA, CDVA, SE, Q value, CCT, Km
Shengtao Liu (25)	English	Prospective	14	SMILE	Allogeneic	29.00 ± 9.17	UDVA, CDVA, SE, CCT, Km
Sheetal Brar (22)	English	Retrospective	42	SMILE	Allogeneic	27.04 ± 5.33	UDVA, CDVA, SE, CCT, Km, Q value, HOAs
Mengfei Hu (27)	Chinese	Retrospective	12	SMILE	Autologous	29.25 ± 5.02	SE, CCT, Km
Yuehua Zhou (9)	Chinese	Prospective	37	SMILE	Allogeneic	28.00 ± 9.00	SE, Km, IOPg, IOPcc, CH, CRF,CCT

SMILE, small incision lenticule extraction; LASIK, laser *in situ* keratomileusis; UDVA, uncorrected distance visual acuity; SE, spherical equivalent; CCT, central corneal thickness; Km, *K* value; CD, corneal densitometry; IOPg, Goldmann correlated intraocular pressure; IOPcc, corneal compensated intraocular pressure; CH, corneal hysteresis; CFR, corneal resistance factor.

### 3.3 Quality assessment

Six of ten prospective single-arm uncontrolled studies (9, 21–23, 25, 26) were evaluated using the MINORs, with a score of 11–13 (Table 2A). The remaining four retrospective uncontrolled studies (10, 20, 24, 27) were evaluated using the JBI case series key assessment checklist and met 9 or more of the 10 criteria (Table 2B). The quality of the included studies met these criteria.

**Table 2:** Quality assessment of included studies.

(A) MINORS index for included non-randomized studies									
Study	I	II	III	IV	V	VI	VII	VIII	Total
Meng Li (23)	2	2	2	2	1	2	2	0	13
Jiawei Wu (21)	2	2	2	2	1	2	2	0	13
Ling Sun (24)	2	2	2	2	1	2	2	0	13
Shengtao Liu (26)	2	2	2	2	0	2	2	0	12
Shengtao Liu (25)	2	2	2	2	0	2	2	0	12
Yuehua Zhou (9)	2	2	2	2	1	2	2	0	13

(B) JBI critical appraisal checklist for case series for included retrospective studies											
Study	Q1	Q2	Q3	Q4	Q5	Q6	Q7	Q8	Q9	Q10	Overall appraisal
Jing Zhang (10)	Yes	Yes	Yes	Yes	Yes	Yes	Yes	Yes	No	Yes	Include
Jie Hou (20)	Yes	Yes	Yes	Yes	Yes	Yes	Yes	Yes	No	Yes	Include
Sheetal Brar (22)	Yes	Yes	Yes	Yes	Yes	Yes	Yes	Yes	No	Yes	Include
Mengfei Hu (27)	Yes	Yes	Yes	Yes	Yes	Yes	Yes	Yes	No	Yes	Include

### 3.4 Outcomes

#### 3.4.1 Visual outcomes

**Figure 2:**
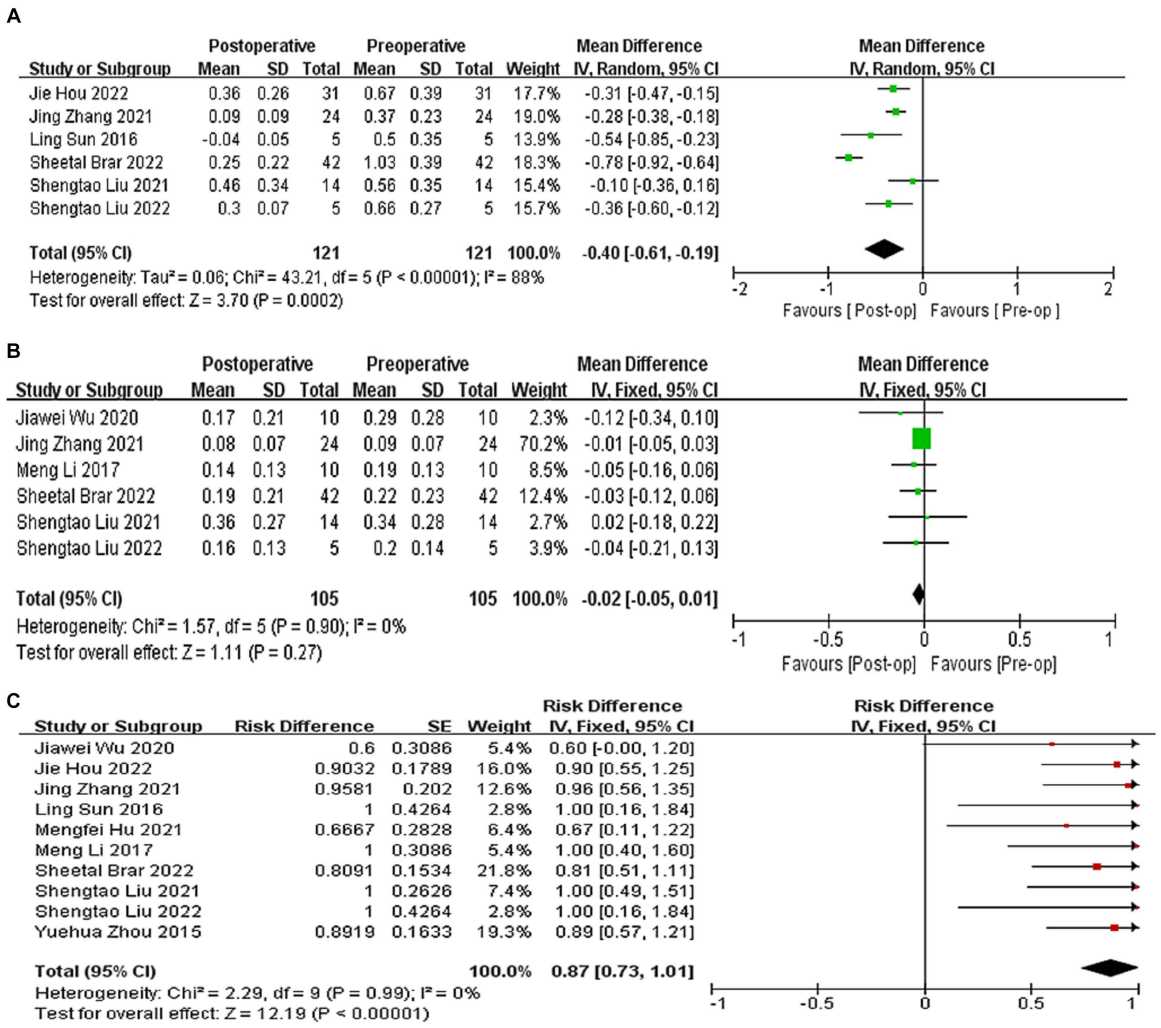
**(A)** Forest plot showing the weighted mean difference of postoperative UDVA (logMAR) and preoperative UDVA (logMAR). **(B)** Forest plot showing the weighted mean difference of postoperative CDVA (logMAR) and preoperative CDVA (logMAR). **(C)** Forest plot showing the risk difference of postoperative CDVA (Snellen).

A total of six studies (9, 20, 22, 24–26) reported the of logMAR values of postoperative UDVA. At the last follow-up, postoperative UDVA increased by 0.40 logMAR compared to preoperative UDVA (WMD = −0.40, 95% CI: [−0.61, −0.19], *I*^2^ = 88%, *p* = 0.0002, Figure 2A). A total six studies (9, 21–23, 25, 26) reported the logMAR values of postoperative CDVA. At the last follow-up, postoperative CDVA increased by 0.02 logMAR compared to preoperative CDVA (WMD = −0.02, 95% CI: [−0.05, −0.01], *I*^2^ = 0%; *p* = 0.27, Figure 2B). All studies reported changes in the Snellen of CDVA. 87% of eyes had no loss of one or more lines in the Snellen lines of the CDVA after surgery (95% CI: [0.73, 1.01], *I*^2^ = 0%, *p* < 0.0001, Figure 2C). Sensitivity analysis showed that, regardless of which article was excluded, the changes in UDVA and CDVA were stable, with no changes in heterogeneity or results.

#### 3.4.2 Refraction outcome

**Figure 3:**
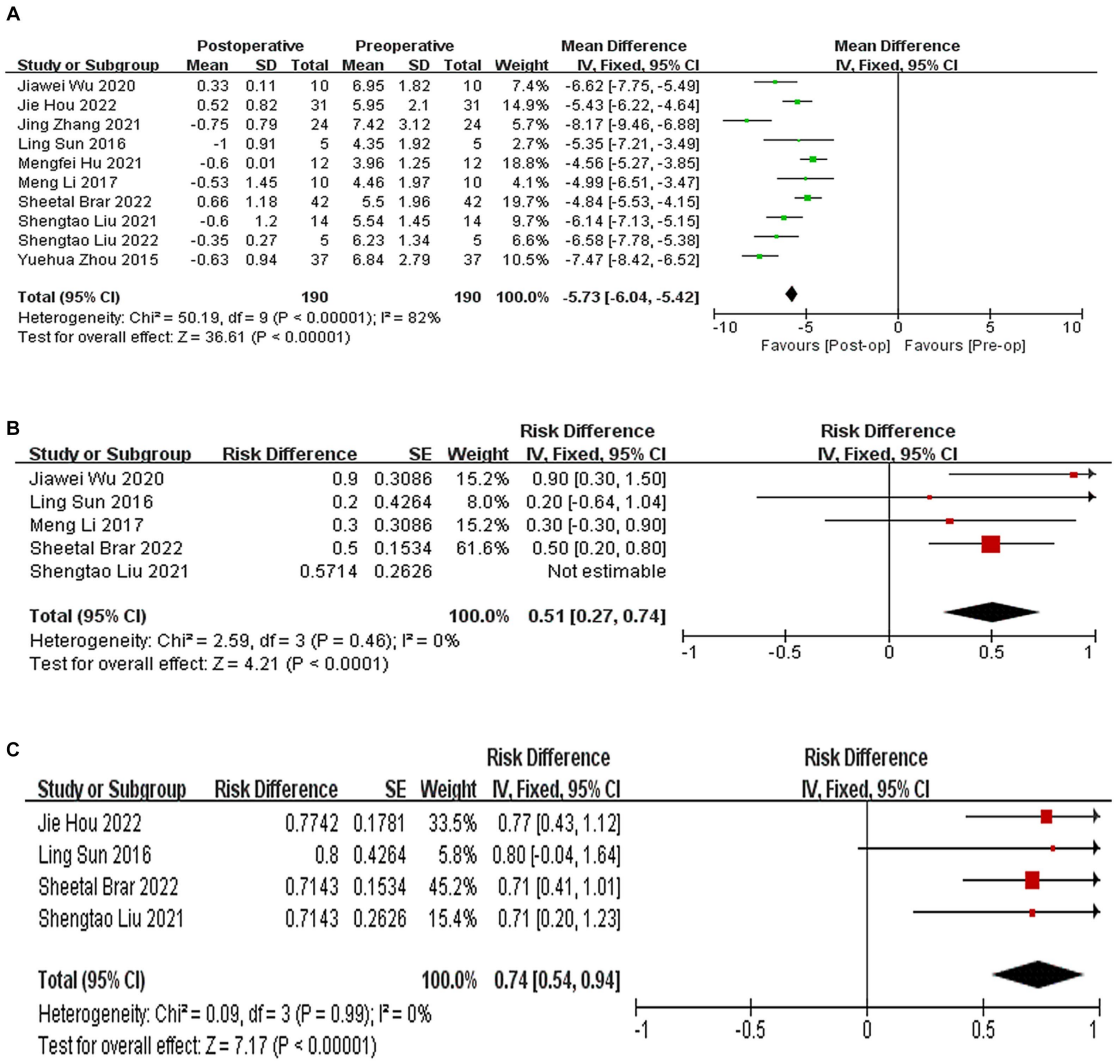
**(A)** Forest plot showing the weighted mean difference of postoperative SE and preoperative SE. **(B)** Forest plot showing the risk difference of postoperative and expected refractive error within the range of ±0.5D. **(C)** Forest plot showing the risk difference of postoperative and expected refractive error within the range of ±1.D.

All 10 studies reported the results of postoperative SE, which decreased by 5.73D compared to preoperative SE, (WMD = −5.73, 95% CI: [−6.04, −5.42], *I*^2^ = 82%, *p* < 0.00001, Figure 3A). A total of 5 studies (21–24, 26) reported the results of the proportion of postoperative and expected refractive error within the range of ±0.5D, and the proportion of postoperative and expected refractive error within the range of ±0.5D was 51% (95% CI: [0.27, 0.74], *I*^2^ = 0%, *p* < 0.0001, Figure 3B). A total of four studies (20, 22, 24, 26) indicated that 74% of the eyes had an error range of ±1.0D compared to the expected SE. (95% CI: [0.54, 0.94], *I*^2^ = 0%, *p* < 0.0001, Figure 3C). Sensitivity analysis shows that regardless of which article is excluded, the changes in the above results are stable, with no heterogeneity or changes in the results.

#### 3.4.3 CCT and corneal curvature

**Figure 4:**
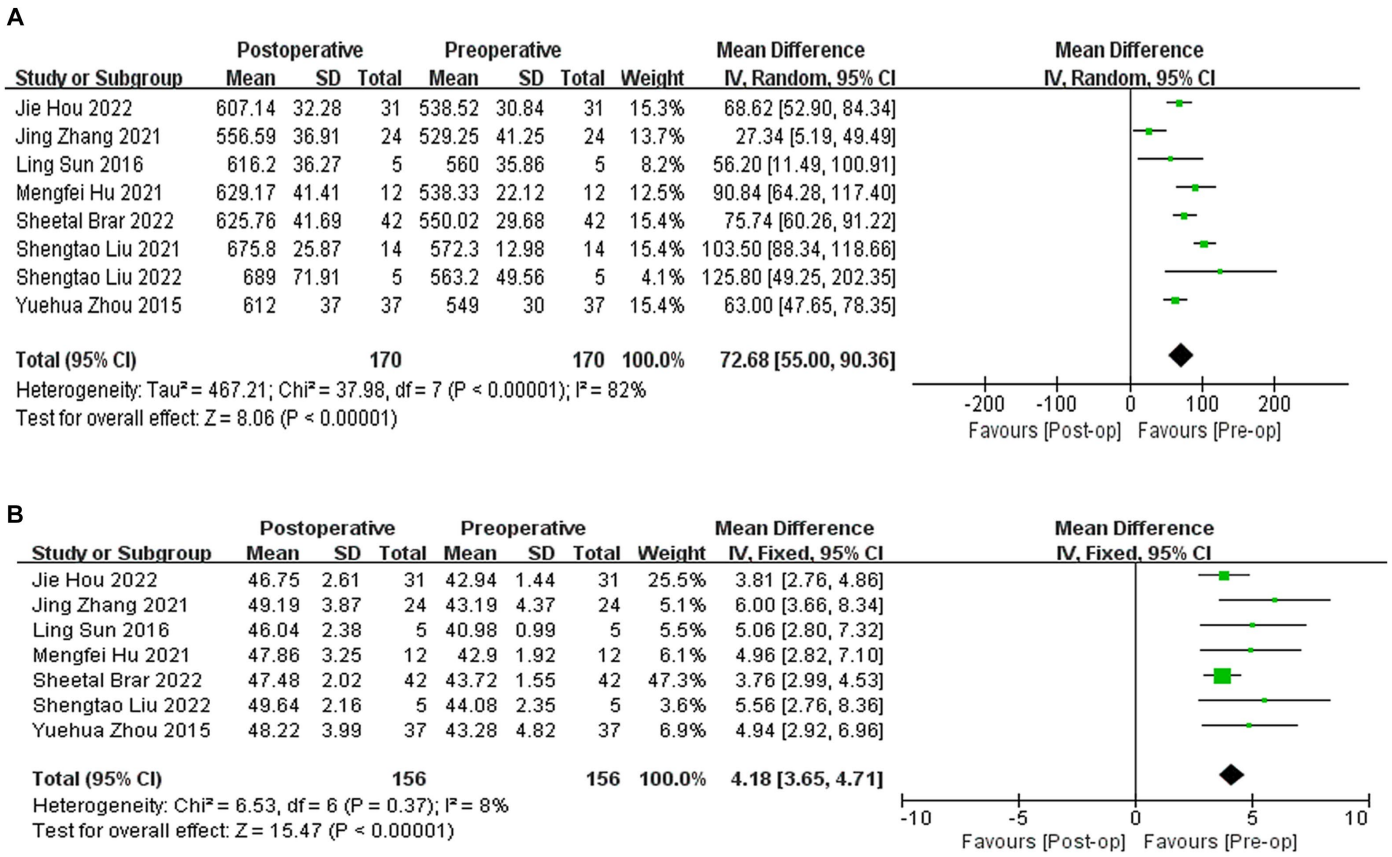
**(A)** Forest plot showing the weighted mean difference of postoperative CCT and preoperative CCT. **(B)** Forest plot showing the weighted mean difference of postoperative corneal curvature and preoperative corneal curvature.

A total of eight studies (9, 10, 20, 22, 24–27) reported the results of CCT, with an average increase of 72.68 μm in postoperative CCT compared to preoperative CCT (WMD = 72.68, 95% CI: [55.00, 90.36], *I*^2^ = 82%; *p* < 0.00001, Figure 4A). Seven studies (9, 10, 20, 22, 24, 25, 27) reported the results of corneal curvature, with an average increase of 4.18D in postoperative corneal curvature compared to preoperative (WMD = 4.18, 95% CI: [3.65, 4.71], *I*^2^ = 8%; *p* < 0.00001,Figure 4B). Sensitivity analysis showed that regardless of which article was excluded, the changes in CCT and corneal curvature were stable, with no changes in heterogeneity or results.

#### 3.4.4 HOAs and *Q*-value

**Figure 5:**
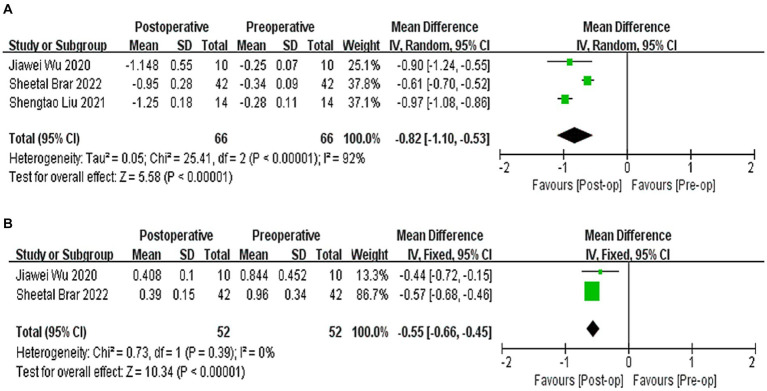
**(A)** Forest plot showing the weighted mean difference of postoperative *Q*-values and preoperative *Q*-values. **(B)** Forest plot showing the weighted mean difference of postoperative HOAs and preoperative HOAs.

Three studies (21, 22, 26) reported the results of *Q*-values, with a difference of −0.82 between postoperative and preoperative *Q*-values, indicating a relative tendency towards swelling (WMD = −0.82; 95% CI: [−1.10, −0.53], *I*^2^ = 90%, *p* < 0.00001, Figure 5A). Two studies (21, 22) reported the results of HOAs, with a difference of −0.55 between postoperative and preoperative HOAs (WMD = −0.55, 95% CI: −[0.66, −0.45], *I*^2^ = 0%, *p* < 0.00001, Figure 5B). Sensitivity analysis showed that regardless of which article was excluded, the changes in *Q*-values and HOAs were stable, with no changes in heterogeneity or results.

#### 3.4.5 Corneal biomechanics

**Figure 6:**
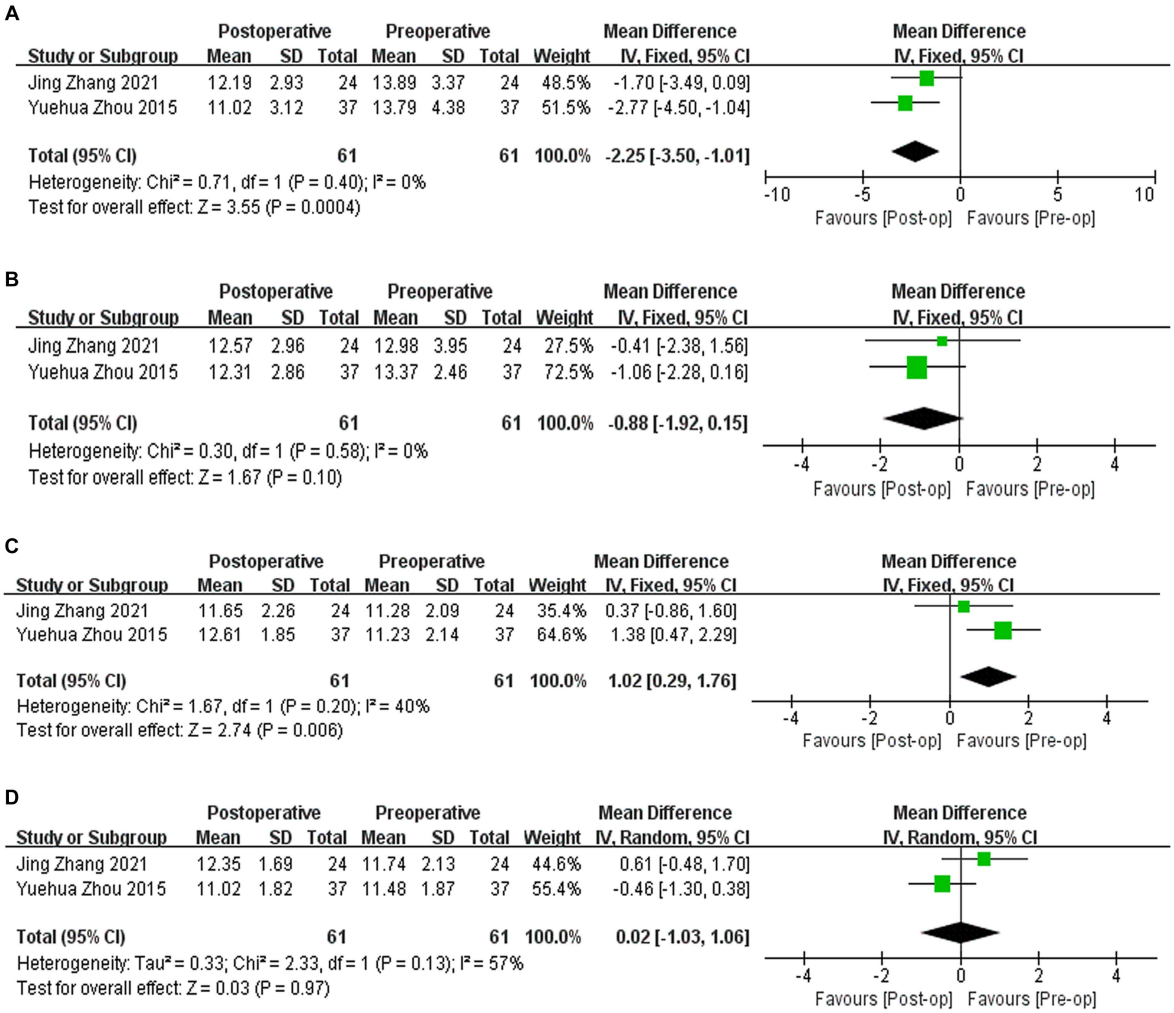
**(A)** Forest plot showing the weighted mean difference of postoperative IOPg and preoperative IOPg. **(B)** Forest plot showing the weighted mean difference of postoperative IOPcc and preoperative IOPcc. **(C)** Forest plot showing the weighted mean difference of postoperative CH and preoperative CH. **(D)** Forest plot showing the weighted mean difference of postoperative CRF and preoperative CRF.

Two studies (9, 10) reported the results of corneal biomechanical indicators, including IOPg, IOPcc, CH, and CRF. IOPg decreased by 2.25 mmHg compared to that before surgery (WMD = −2.25; 95% CI: [−3.50, −1.01]; *I*^2^ = 0%; *p* = 0.0004, Figure 6A). There was no significant difference in IOPcc compared to preoperative values (WMD = −0.88, 95% CI: [−1.92, 0.15], *I*^2^ = 0%; *p* = 0.10, Figure 6B). CH increased by 1.02 compared to the preoperative value (WMD = 1.02, 95% CI: [0.29, 1.96], *I*^2^ = 40%, *p* = 0.006, Figure 6C). There was no significant difference in postoperative CFR compared to preoperative values (WMD = 0.02, 95% CI: [−1.03, 1.06], *I*^2^ = 57%, *p* = 0.97, Figure 6D). Sensitivity analysis shows that regardless of which article is excluded, heterogeneity and results will change.

#### 3.4.6 Sensitivity analysis and publication bias

**Figure 7:**
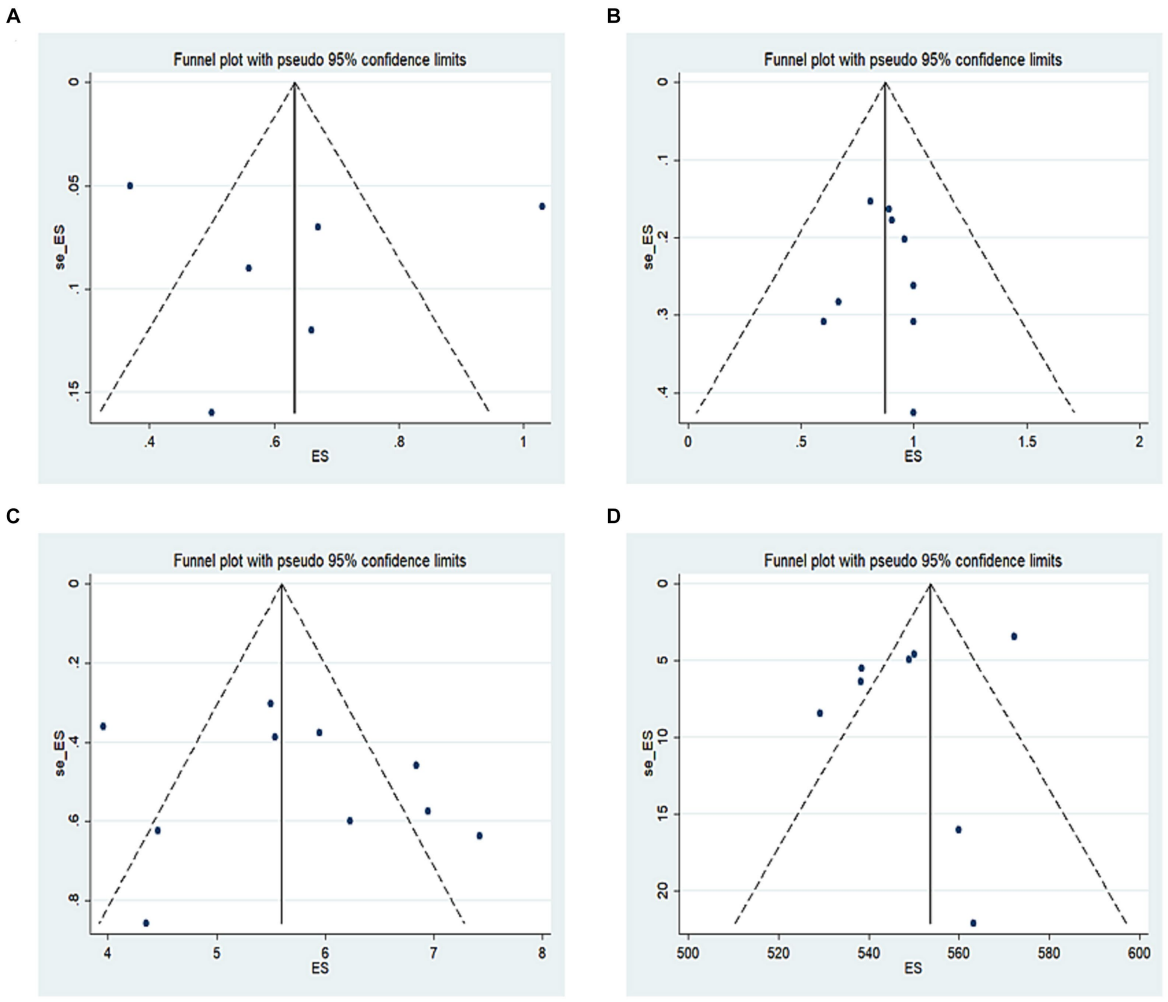
**(A)** Funnel plot of postoperative changes in UDVA, **(B)** funnel plot of postoperative changes in CDVA, **(C)** funnel plot of postoperative changes in SE, **(D)** funnel plot of postoperative changes in CCT.

Publication bias was evaluated intuitively using a funnel plot of postoperative changes in UDVA (Figure 7A), CDVA (Figure 7B), SE (Figure 7C), and CCT (Figure 7D). We also conducted Egger regression to quantitatively evaluate publication bias, and found that *p* (UDVA) = 0.54, *p* (CDVA) = 0.81, *p* (SE) = 0.49, and *p* (CCT) = 0.31, indicating that the funnel plot was symmetric, and there was no publication bias in this study.

## 4 Discussion

This study included 10 studies with a total of 130 patients (190 eyes). The meta-analysis results showed that the postoperative UDVA of corneal intrastromal lenticule implantation for hyperopia was significantly increased. The proportion of postoperative CDVA that reached or surpassed the preoperative CDVA was 87%. The proportion of postoperative SE error within ±0.5D was 52%, and the proportion of postoperative SE error within ±0.1.0D was 74%. The above results suggest that the application of corneal intrastromal lenticule implantation to correct hyperopia can effectively improve the refractive state of patients and achieve correction of refractive errors. Although LASIK surgery can also achieve the effect of correct hyperopia, significant refractive regression was observed in long-term follow-up studies (28, 29). Studies by Zhang (10), Li (23), and Sheetal (22) were followed up for 1 year, 2 years, and 3 years (1–7 years) after surgery, all showing good refractive stability.

The difference in postoperative *Q*-value compared to the preoperative *Q*-value was −0.82, which is directly related to the implantation of corneal intrastromal lenticule, leading to a relative tendency of the cornea to bulge. After surgery, HOAs decreased by 0.55 compared to before surgery. In theory, the smaller the eccentricity of the optical region center, the lower the introduction of higher-order aberrations (30). SMILE surgery is superior to LASIK in this regard (31, 32). Different types of corneal refractive surgery can cause changes in the *Q*-value (33). Currently, *Q*-value guided LASIK surgery can be performed, and a large amount of clinical data has achieved good results (34, 35). However, this issue needs to be addressed further in the treatment of hyperopia with corneal intrastromal lenticule implantation.

The average increase in postoperative CCT compared to preoperative was 72.68 μm. After surgery, the corneal curvature increased by an average of 4.18D compared to before surgery. Corneal intrastromal lenticule implantation increases corneal thickness and effectively preserves corneal stromal thickness, avoiding the risk of corneal dilation (36). Postoperative corneal topography shows that the central part of the cornea has become significantly convex, with increased curvature, which changes the refractive power of the central part of the cornea and corrects hyperopia. At the same time, this new surgical method for correcting hyperopia avoids the possibility of passive formation of excessively high central curvature of the cornea and does not lead to surgically induced KC. In addition, changing corneal morphology through implantation is a reversible surgical approach when there are other diseases that require feasible lenticule removal surgery. In our meta-analysis, the results of the two included studies were inconsistent, and convincing and consistent evidence regarding corneal biomechanical indicators has not yet been found, which deserves further validation through case-control studies.

The corneal stroma accounts for 90% of the corneal thickness, which is crucial for ensuring the corneal transparency and refractive function necessary for normal vision. Currently, scientific research on corneal stroma mainly includes acellular or decellularized and decellularized human or animal corneas and non-corneal tissues, acellular bioengineered stromal scaffolds, tissue adhesives, 3D bioprinting, and stromal stem cell therapy (37, 38). As of 2022, the global surgical volume of SMILE has exceeded 6 million cases, generating a large number of corneal stromal lenses annually. Currently, attempts have been made to apply corneal intrastromal lenticule for the treatment of diseases such as hyperopia correction, ulcerative keratitis, KC, and corneal dilation after LASIK; however, they have not yet achieved widespread clinical application. When using corneal intrastromal lenticule implantation to treat hyperopia, the corneal stroma tissue implanted in the capsule has an ordered arrangement of collagen fibers, with a lens diameter of 6.5 mm, without blood vessels or lymphatic tissue. The corneal stroma capsule is in a sterile environment, and the donor corneal stroma is not in contact with aqueous humor or tears. Therefore, the probability of corneal graft rejection and corneal infection is very low. However, in Brar’s study (22), four eyes experienced immune rejection, all of which were cryopreserved. The use of fresh corneal intrastromal lenticules can also improve graft survival and reduce the rejection rate (39). At present, research has achieved non-traditional cryopreservation of corneal stromal lenses, improving their activity and meeting the needs of corneal intrastromal lenticule implantation (40–42).

### 4.1 Study limitations

Through literature review and meta-analysis, we found that there are few studies on the treatment of hyperopia with corneal intrastromal lenticule implantation, and the research design has certain limitations. The number of included research cases was small, and due to the limited number of cases, it was not possible to perform subgroup analysis of corneal intrastromal lenticule implantation surgery and autologous and allogeneic transplantation. In our actual clinical work, while paying attention to postoperative visual acuity recovery, we should also pay attention to the long-term safety and changes in corneal morphology after surgery. There is relatively little research on corneal biomechanics and corneal curvature, and the research results have a certain degree of difference. In-depth research can be conducted in future clinical studies to address this issue, providing more sufficient evidence for the safety and effectiveness of corneal intrastromal lenticule implantation in the treatment of hyperopia.

## 5 Conclusion

Corneal intrastromal lenticule implantation surgery can effectively improve the refractive state of patients with hyperopia, improve their vision, and reduce the risk of postoperative corneal dilation and keratoconus caused by corneal refractive surgery, achieving the reversibility of corneal refractive surgery.

## Data availability statement

The original contributions presented in the study are included in the article/supplementary material, further inquiries can be directed to the corresponding author.

## Author contributions

YW: Formal analysis, Resources, Writing – original draft. JZ: Formal analysis, Writing – review & editing. ZG: Project administration, Writing – review & editing. XF: Funding acquisition, Visualization, Writing – original draft.
